# TOT3–AHA1 module: its role in fine-tuning stomatal responses

**DOI:** 10.3389/fpls.2025.1582196

**Published:** 2025-05-29

**Authors:** Lei Gong, Nadiyah M. Alabdallah, Faizah Amer Altihani, Siham M. AL-Balawi, Hanan Khalaf Anazi, Basmah M. Alharbi, Md. Mahadi Hasan

**Affiliations:** ^1^ School of Agriculture and Bioengineering, Longdong University, Qingyang, China; ^2^ Department of Biology, College of Science, Imam Abdulrahman Bin Faisal University, Dammam, Saudi Arabia; ^3^ Basic and Applied Scientific Research Centre, Imam Abdulrahman Bin Faisal University, Dammam, Saudi Arabia; ^4^ Biology Department, Faculty of Science, King Khalid University, Abha, Saudi Arabia; ^5^ Biology Department, Faculty of Science, University of Tabuk, Tabuk, Saudi Arabia; ^6^ Biodiversity Genomics Unit, Faculty of Science, University of Tabuk, Tabuk, Saudi Arabia; ^7^ State Key Laboratory of Herbage Improvement and Grassland Agro-ecosystems, College of Ecology, Lanzhou University, Lanzhou, China

**Keywords:** abscisic acid, carbon di-oxide, guard cell, light, photosynthesis

## Abstract

Stomatal pores, controlled by surrounding guard cells, play a crucial role in balancing carbon dioxide (CO_2_) uptake and water loss in plants. Recent studies in *Arabidopsis thaliana* have identified the TARGET OF TEMPERATURE 3 (TOT3)–plasma membrane (PM) H^+^-ATPase (AHA1) module as a key player in stomatal movement, though its exact role in vascular plants is not yet fully understood. TOT3, a transcriptional regulator, influences the activity of AHA1, which regulates ion fluxes essential for stomatal opening. Under high-temperature conditions, TOT3 promotes stomatal opening, while OST1, a key regulator of drought-induced stomatal closure, inactivates TOT3 through phosphorylation. This phosphorylation serves as a molecular switch, adjusting stomatal aperture in response to heat and drought stress. Moreover, light may also impact stomatal movement via the TOT3–AHA1 interaction. This review explores the molecular mechanisms underlying the TOT3–AHA1 module, its integration with abscisic acid (ABA) signaling, and its role in plant adaptation to environmental stresses. Understanding this pathway could contribute to developing crops with enhanced water-use efficiency and resilience to abiotic stress.

## 1 Introduction

Stomata, formed by guard cells in the epidermis, regulate gas exchange by balancing water loss and photosynthesis in response to environmental cues like light, humidity, and CO_2_ (Inoue and Kinoshita, 2017). Their opening enhances carbon di-oxide (CO_2_) uptake, transpiration, and nutrient transport, impacting plant growth, yield, and water-use efficiency (Wang et al., 2014; Zhang et al., 2021). In response to drought and heat stress, plants initiate cellular and physiological adaptations to sustain function (Ando et al., 2022). Among these responses, stomatal opening and closing occur rapidly, likely requiring switch-like signaling mechanisms to adapt to fluctuating environmental conditions. Kinase-mediated phosphorylation, being fast and reversible, serves as an efficient regulatory mechanism for dynamic stomatal responses (Kholodenko and Okada, 2021).

Drought stress leads to dehydration and wilting, prompting stomatal closure to reduce water loss (Hasan et al., 2021, 2024). This response is driven by ABA, which is synthesized under water-deficit conditions and activates ABA signaling in guard cells to regulate ion channels, facilitating stomatal closure (Hasan et al., 2021). In contrast, plasma membrane (PM) H^+^-ATPase (AHA) ion pumps play a key role in stomatal opening by extruding protons and modulating membrane potential (Toh et al., 2021). In *Arabidopsis thaliana*, activation of AHA occurs via phosphorylation of the Thr947/948 residue, which creates a binding site for 14-3–3 proteins, relieving autoinhibition (Hayashi et al., 2024; Falhof et al., 2016). However, ABA inhibits AHA activity, as seen in *ost2-1D* and *ost2-2D* mutants, which exhibit ABA insensitivity due to a constitutively active AHA1 allele (Hayashi et al., 2011; Miao et al., 2022). High ABA concentrations reduce AHA1 activity by promoting Thr947 dephosphorylation, leading to stomatal closure (Hayashi et al., 2011; Falhof et al., 2016; Miao et al., 2022). The ABA-responsive kinase OPEN STOMATA 1 (OST1), expressed in guard cells, regulates ion transporters and controls stomatal closure under drought stress (Belin et al., 2006; Hasan et al., 2021). While OST1 is implicated in repressing AHA activity, the precise mechanism remains unclear (Hayashi et al., 2011).

Conversely, high-temperature stress promotes stomatal opening and increased conductance, which lowers leaf temperature (Sinha et al., 2022). In *Arabidopsis*, phototropins (PHOT1 and PHOT2), activated by blue light, contribute to this cooling effect by phosphorylating BLUE LIGHT SIGNALLING1 (BLUS1) and MAP4K10, which in turn activate PM H^+^-ATPase to drive stomatal opening (Kostaki et al., 2020). Notably, stomatal opening under high temperatures also occurs in darkness, indicating the presence of additional regulatory mechanisms (Kostaki et al., 2020). The kinase TARGET OF TEMPERATURE 3 (TOT3)/MAP4K4 regulates stomatal opening at high temperatures by phosphorylating PM H^+^-ATPases, while OST1 inactivates TOT3 under drought conditions to conserve water (Xu et al., 2024). This phosphorylation-based mechanism highlights MAP4Ks as key regulators of stomatal aperture under abiotic stress (Hayashi et al., 2024). Light also plays a crucial role in stomatal movement by activating ion transporters in guard cells, increasing turgor pressure for pore expansion. The TOT3–AHA1 module likely modulates AHA1 activity, linking light signals to ion homeostasis, with blue and red-light pathways governing stomatal opening. The identification of the TOT3–AHA1 module offers new insights into stomatal regulation. This review discusses how TOT3 interacts with AHA1 to control stomatal behavior and explores its integration with environmental signals (drought, heat, and light) and guard cell signaling in plant physiology. These discussions are primarily based on findings from *Arabidopsis thaliana*, a model system that has provided foundational insights into stomatal regulation, with implications for broader plant species.

## 2 Insights into the TOT3–AHA1 module

### 2.1 TOT3: a transcriptional regulator of stomatal function

TOT3 is a transcription factor crucial for stomatal function, influencing guard cell physiology by regulating ion transporter expression and signaling molecules (Xu et al., 2024; Praat et al., 2021). Vu et al. (2021) demonstrated that MAP4K4/TOT3 interacts with related kinases MAP4K6/TOI4 and MAP4K5/TOI5, which also contribute to thermomorphogenesis. Similar interactions among closely related kinases have been observed in receptor-like kinases involved in ligand perception and phosphorylation. A comparable case is the OPEN STOMATA 1 (OST1)/SnRK2.6 complex, which interacts with SnRK2.2, SnRK2.3, and SnRK2.8 to enhance SnRK2 signaling under salt or osmotic stress (Vu et al., 2021; Praat et al., 2021). While these kinases share functional redundancy, they also exhibit distinct roles. In mammals, MAP4Ks also form homo- and heterodimers with specific functions. The thermomorphogenic phenotypes of *tot3 toi4* and *tot3 toi5* double mutants, and especially the *tot3 toi4 toi5* triple mutant, suggest functional overlap among TOT3, TOI4, and TOI5 (Kerbler and Wigge, 2023). However, the absence of a clear phenotype in the *toi4 toi5* double mutant at high temperatures highlights TOT3’s dominant role in thermal response regulation.

### 2.2 AHA1: the plasma membrane H^+^- ATPase

The plasma membrane (PM) H^+^-ATPase is a crucial ion pump in plant cells, responsible for expelling protons to generate membrane potential, which is essential for growth. Its activity is regulated by modifications to its autoinhibitory terminal domains, suggesting these regions serve as targets for physiological factors that modulate proton pumping (Falhof et al., 2016). This ATPase is encoded by a multigene family with multiple isoforms, including 11 in *Arabidopsis thaliana* (AHA1–AHA11) (Palmgren, 2001). Among them, *AHA1* and *AHA2* are widely expressed in plant tissues, while *AHA10* is specifically linked to seed development (Harper et al., 1994). AHA1, a key proton pump in the guard cell plasma membrane, generates the electrochemical gradients necessary for ion transport. Its activation causes membrane hyperpolarization, promoting K^+^ uptake, guard cell expansion, and ultimately, stomatal opening. Recent findings suggest that TOT3 indirectly affects AHA1 activity by influencing its transcriptional regulation or post-translational modifications. It may also participate in regulating kinases or phosphatases that govern AHA1 function.

## 3 Signaling pathways integrating the TOT3–AHA1 module

### 3.1 ABA and stomatal closure

Stomatal closure is regulated through both active mechanisms (ABA-mediated signaling) and passive mechanisms (hydraulic responses). In the ABA-dependent pathway, a reduction in guard cell turgor leads to stomatal closure, primarily through the inhibition of H^+^-ATPase activity and the activation of anion channels (Doi et al., 2015). While the precise role of TOT3 in ABA signaling remains unclear, it is proposed to function as a key regulatory node that links ABA perception to the expression and activity of ion transporters. Recent research has shed light on the TOT3–AHA1 module, revealing its involvement in ABA-driven stomatal regulation. This module appears to play a crucial role in fine-tuning guard cell responses, contributing to the intricate molecular mechanisms that optimize stomatal function under varying environmental conditions.

ABA functions as a central hub that integrates multiple signaling networks to regulate stomatal closure (Hasan et al., 2021). The core ABA signaling pathway consists of ABA receptors, phosphatases (PP2Cs), and kinases (SnRK2s), which coordinate downstream responses (Nishimura et al., 2010). Among these, ABI1 and ABI2, encoding PP2C proteins, regulate the phosphorylation and activation of SnRK2 kinases, including SnRK2.2/D, SnRK2.3/E, and SnRK2.6/OST1/E, which are key mediators of ABA responses (Hasan et al., 2021). A critical event in this pathway is the regulation of ion fluxes that drive stomatal movement. ABA-mediated activation of SnRK2s leads to the phosphorylation of S-type anion channels such as SLAC1 and its homolog SLAH3, enabling Cl^-^ and NO_3_^-^ efflux (Roelfsema et al., 2012). The *slac1* mutant shows an impaired response to ABA and Ca²^+^-induced stomatal closure (Vahisalu et al., 2008). Activation of SLAC1 results in plasma membrane (PM) depolarization, which in turn activates GORK, an outward-rectifying K^+^ channel (Hasan et al., 2022a). The efflux of K^+^ through GORK reduces turgor pressure in guard cells, ultimately causing stomatal closure.

**Figure 1:**
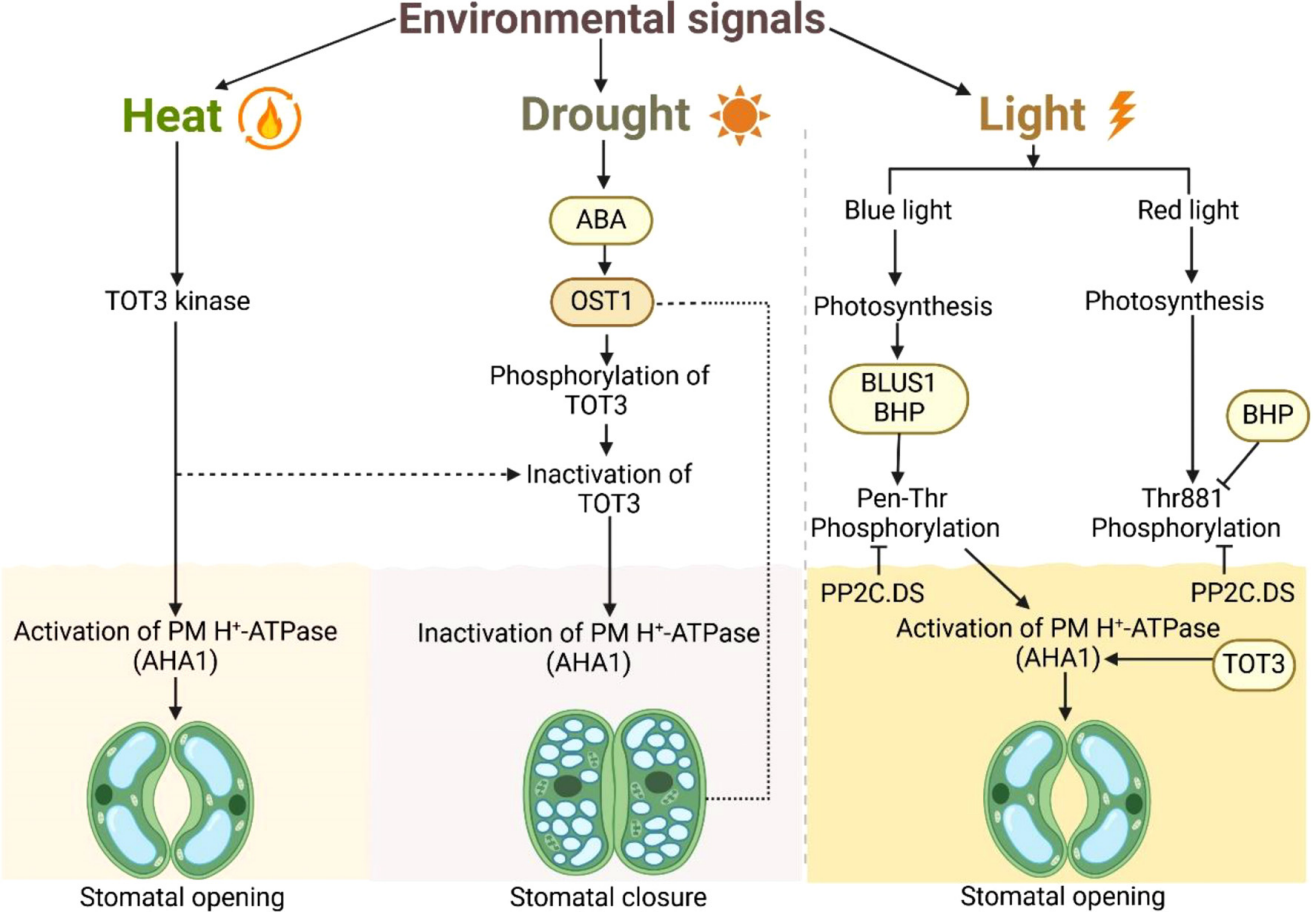
Proposed model of AHA1–OST1–TOT3 module-regulated stomatal aperture modulation under heat, drought stress and light signaling. The diagram illustrates the effects of heat, drought, and light on stomatal regulation in plants. Heat triggers TOT3 kinase, which activates plasma membrane H^+^-ATPase (AHA1), leading to stomatal opening. Drought induces abscisic acid (ABA) signaling, activating OST1, which phosphorylates and inactivates TOT3, ultimately resulting in stomatal closure due to PM H^+^-ATPase (AHA1) inactivation. Light regulates stomatal movement via blue and red light signaling. Blue light induces phosphorylation of BLUS1 and BHP, activating PP2C.DS, which in turn activates PM H^+^-ATPase (AHA1), promoting stomatal opening. Similarly, red light-induced photosynthesis leads to the phosphorylation of BHP at Thr881, activating PP2C.DS and facilitating the activation of PM H^+^-ATPase (AHA1). The dashed line represents the indirect inhibition of AHA1 during drought.

Within this regulatory framework, TOT3 and AHA1 play a crucial role in fine-tuning membrane polarization and ion fluxes. TOT3 is a key regulatory protein that interacts with various components of the cytoskeleton (microtubules, actin filaments) and signaling pathways (ABA, Ca^2+^, light dependent signaling pathways etc.) involved in stomatal movement (**Figure 1**).

On the other hand, AHA1, a plasma membrane H^+^-ATPase, plays a central role in proton transport, generating membrane potential and modulating guard cell turgor pressure (Yamauchi et al., 2016). TOT3 is proposed to modulate AHA1, a plasma membrane H^+^-ATPase, which directly influences proton transport and membrane potential (Xu et al., 2024). Under ABA signaling, the inhibition of AHA1 reduces proton extrusion, promoting depolarization of the guard cell plasma membrane (Merlot et al., 2007). This depolarization enhances the activity of anion channels like SLAC1, facilitating anion efflux, which drives K^+^ efflux through GORK (Hasan et al., 2021). The net loss of ions and the subsequent osmotic water loss lead to guard cell shrinkage and stomatal closure. Furthermore, the interaction between TOT3 and AHA1 is likely subject to post-translational modifications and feedback regulation by ABA-responsive kinases and phosphatases (Xu et al., 2024). This dynamic control ensures that stomatal responses are precisely regulated in response to environmental fluctuations, optimizing plant water use efficiency. Future studies unraveling the precise regulatory mechanisms and identifying additional interacting partners could provide potential targets for improving drought resistance in crops through biotechnological or genetic approaches.

### 3.2 CO_2_ and environmental stress responses (drought, and heat)

Stomatal aperture in plants is primarily controlled by environmental factors such as elevated CO_2_ levels, drought, and heat stress to balance gas exchange and water use efficiency. The TOT3–AHA1 module plays a crucial role in integrating these environmental cues to regulate stomatal movement, aiding plant adaptation to fluctuating conditions. Stomata close when CO_2_ levels rise and open when they decrease. Elevated CO_2_ triggers the activation of the SLOW ANION CHANNEL-ASSOCIATED 1 (SLAC1), leading to rapid stomatal closure (Vahisalu et al., 2008). CO_2_ enters guard cells via the PLASMA MEMBRANE INTRINSIC PROTEIN 2 (PIP2) channel or through transmembrane diffusion (Wang et al., 2016). This process involves the activation of HT1 kinase, MPK4/MPK12, and SLAC1, facilitating ion efflux from guard cells (Hõrak et al., 2016; Jakobson et al., 2016). The TOT3–AHA1 module is believed to modulate this response by regulating ion and proton homeostasis. By influencing AHA1 activity, TOT3 likely promotes guard cell depolarization, facilitating the efflux of Cl^-^, NO_3_^-^, and K^+^, ultimately leading to stomatal closure.

Apart from CO_2_, drought and heat stress also impact stomatal function. Under drought, ABA promotes stomatal closure and inhibits reopening to minimize water loss (Hasan et al., 2021). ABA, along with hydrogen peroxide (H_2_O_2_) and nitric oxide (NO), activates the MAPK signaling pathway, increasing ABA levels and triggering SnRK2 kinase activation (Hasan et al., 2021), which can suppress AHA1 activity. The TOT3–AHA1 interaction enables rapid proton flux adjustments, leading to membrane depolarization, anion channel activation, and stomatal closure. In contrast, high temperatures promote stomatal opening to facilitate cooling (Xu et al., 2024). However, simultaneous exposure to heat and drought creates conflicting stomatal responses, and the underlying regulatory mechanisms remain unclear. The high-temperature-responsive kinase TOT3 regulates plasma membrane H^+^-ATPases to induce stomatal opening (Xu et al., 2024). While TOT3 activity increases with heat stress, it is suppressed when heat and drought occur together. During drought, OPEN STOMATA 1 (OST1) phosphorylates and inactivates TOT3, reinforcing stomatal closure (Xu et al., 2024). Understanding how environmental stress signals interact in stomatal regulation is essential for developing crops resilient to climate change.

### 3.3 Light and stomatal opening

Light is a crucial environmental factor that significantly impacts plant growth and reproduction by regulating various physiological processes, including stomatal movement (Hasan et al., 2022b). Stomatal opening is primarily controlled by light signals, which activate ion transporters in guard cells, leading to increased turgor pressure and pore expansion. The H^+^-ATPase (AHA1) plays a key role in this process by creating a proton gradient that facilitates ion and water uptake into guard cells, promoting stomatal opening. The TOT3–AHA1 module may serve as a regulatory component that modulates AHA1 activity, linking external light signals to ion homeostasis in guard cells. Light-induced stomatal opening is primarily governed by blue and red-light signaling pathways (Li et al., 2022), which are closely associated with the TOT3–AHA1 regulatory mechanism.

#### 3.3.1 Blue light signaling pathway

Red and blue light regulate stomatal opening through distinct mechanisms: red light promotes stomatal opening via photosynthesis, while blue light directly activates plasma membrane (PM) H^+^-ATPases (Kinoshita and Hayashi, 2011). In *Arabidopsis*, blue light is perceived by phototropins (PHOT1 and PHOT2), which contain N-terminal photosensory and C-terminal kinase domains (Christie, 2007). Upon blue light excitation, the LOV2 domain undergoes conformational changes, leading to the autophosphorylation of phototropins and the activation of Blue Light Signaling 1 (BLUS1), a key protein kinase in this pathway (Takemiya et al., 2013). Activated BLUS1 phosphorylates an unknown kinase that targets the penultimate Thr of PM H^+^-ATPases, creating a binding site for 14-3–3 proteins, which further enhances proton pump activity (Falhof et al., 2016). Small auxin upregulated RNAs (SAURs) also contribute by inhibiting PP2C and promoting H^+^-ATPase activation (Ren et al., 2018). Additional regulators include BHP kinase, which acts downstream of *BLUS1*, and *CBC1/CBC2*, which mediate blue light-dependent phosphorylation of H^+^-ATPases (Hiyama et al., 2017). ABA counteracts blue light signaling by promoting H_2_O_2_-induced dephosphorylation of PM H^+^-ATPases, thereby inhibiting stomatal opening (Zhang et al., 2004). However, the precise mechanisms by which BLUS1 directly regulates PM H^+^-ATPases remain unclear, necessitating further investigation (Wong et al., 2021).

#### 3.3.2 Red light signaling pathway

Red light has been shown to induce phosphorylation of PM H^+^-ATPases, correlating with stomatal opening (Ando and Kinoshita, 2018). Library-based chemical screens have identified nine compounds (SCL1–SCL9) that suppress light-induced stomatal opening, particularly in Benghal dayflower (*Commelina benghalensis*) (Kinoshita et al., 2021). Among these, stomatal closing compound 1(SCL1) and stomatal closing compound 2 (SCL2) inhibit blue light-induced phosphorylation at the Thr-947 residue of PM H^+^-ATPases, thereby preventing stomatal opening (Toh et al., 2018). Further screening of protease inhibitors (PIs) revealed three inhibitors (PI1, PI2, and PI3) that block blue light-dependent phosphorylation of PM H^+^-ATPases (Wang et al., 2021). Application of SCL1 or PI1 on leaves effectively reduces stomatal opening and water loss, suggesting potential applications in agriculture to conserve water and extend the shelf life of cut flowers (Toh et al., 2018; Kinoshita et al., 2021). Apart from light quality, light intensity also influences PM H^+^-ATPase activity by altering its localization. 3D live-cell imaging revealed that under dim light, *Arabidopsis* AHA2 accumulates in intracellular compartments, a process regulated by FERONIA (FER) receptor kinase, leading to apoplast alkalization and inhibited root growth (Haruta et al., 2018). In summary, The TOT3–AHA1 module is essential for integrating light signals to regulate stomatal movement, working alongside the ABA signaling pathway, which inhibits plasma membrane H^+^-ATPases (AHA1) to induce closure. TOT3 may act as a scaffold protein, supporting AHA1 activation under blue light and facilitating its phosphorylation, thereby enhancing proton pump activity and promoting ion uptake in guard cells. This dynamic regulation ensures effective proton extrusion and K^+^ uptake, enabling sustained stomatal opening under light conditions.

## 4 Applications of TOT3–AHA1 module in plant science research

### 4.1 Crop improvement strategies

Recent advancements in breeding techniques have provided valuable insights into the molecular basis of abiotic stress tolerance and have contributed to enhancing stress resilience in key crops. Efficient control of stomatal size and regulation of stomatal aperture under drought conditions are crucial for reducing water loss and maintaining optimal photosynthesis, ultimately leading to higher yields (Wang and Chang, 2024). Using a leaf imprinting technique, Shahinnia et al. (2016) performed rapid, non-destructive phenotyping to explore genetic variation and map QTLs associated with stomatal characteristics in wheat. Thus, breeding programs targeting the TOT3–AHA1 module offer a promising strategy to develop drought-tolerant crop varieties with improved water-use efficiency. Combining QTL mapping with high-throughput phenotyping can further accelerate the selection of resilient cultivars, translating *Arabidopsis*-derived insights into improved crop productivity in water-limited environments.

### 4.2 Biotechnology approaches

Modifying stomatal development genes through genetic engineering is an effective method for increasing crop tolerance to stress. For example, overexpressing *EPF1, EPF2, SDD1*, and *TLFP8* has been found to reduce stomatal density, which in turn enhances dehydration resistance in crops such as rice, wheat, barley, maize, and tomato (Lu et al., 2019; Li et al., 2020). Additionally, the CRISPR-Cas9 genome editing system is extensively used to study gene functions related to crop stress responses and improve stress resilience (Gao, 2021). Further progress in CRISPR/Cas-based editing and transcriptomics will be valuable in deciphering the regulatory interactions between TOT3 and AHA1.

### 4.3 Conservation and application potential of the TOT3–AHA1 module in crop species

To effectively translate knowledge of the TOT3–AHA1 module into crop improvement programs, it is essential to first assess the conservation of this regulatory module across agriculturally important species. While the TOT3–AHA1 module has been well characterized in *Arabidopsis thaliana*, its conservation and functional relevance in crop plants remain unexplored. Genomic and transcriptomic studies have identified orthologs of TOT3 and AHA1 in major crops such as wheat and rice (Vu et al., 2021; Toda et al., 2016). For example, *MAP4K4* in wheat, an ortholog of *TOT3*, is upregulated under heat stress, indicating potential functional conservation in thermomorphogenesis (Vu et al., 2021). Similarly, *OsAHA1* in rice shares high sequence similarity with *Arabidopsis* AHA1 and is expressed in guard cells, suggesting a conserved role in stomatal regulation (Toda et al., 2016). Functional studies in crops are limited, and differences in guard cell morphology may affect TOT3–AHA1 function. CRISPR-based editing and transcriptomic analyses are needed to validate their conservation and role in stomatal regulation, aiding the translation of *Arabidopsis* insights to improve water-use efficiency and drought tolerance in cereals.

## 5 Research gap and future direction

In recent years, research on stomatal regulation via the TOT3–AHA1 module has been limited. As a result, several critical questions remain unanswered and require further investigation. For instance, the specific post-translational modifications of AHA1 and their impact on its activity are still not well understood. Additionally, the downstream effectors of the TOT3–AHA1 module beyond ion transport remain unidentified. In addition, it is crucial to explore whether targeted genetic engineering of this module can enhance stress resilience in plants without adversely affecting growth. Bridging these research gaps will require an interdisciplinary approach that integrates genetics and systems biology. Future studies should prioritize investigating the molecular interactions between TOT3 and AHA1 in stomatal regulation. Additionally, the role of the TOT3–AHA1 module in coordinating plant responses to multiple environmental stresses, including drought, salinity, heat, low temperature, and light stress, remains to be explored. Furthermore, comparative studies across *Arabidopsis* and crop species, such as rice and wheat, will elucidate the modules conservation and facilitate its application in agriculture. Finally, long-term field trials and assessments of its potential applications in crop improvement programs will be essential to translating these findings into practical agricultural benefits.

## 6 Conclusion

In conclusion, the TOT3–AHA1 module plays a crucial role in regulating stomatal opening and closing by fine-tuning guard cell responses to environmental and hormonal cues. As a conserved signaling pathway, it modulates AHA1 activity through post-translational modifications, directly influencing stomatal aperture and enabling plants to adapt quickly to environmental changes. Further research on this module may lead to innovative approaches for enhancing plant productivity and stress tolerance.

## References

[B1] Ando E. Kinoshita T. (2018). Red light-induced phosphorylation of plasma membrane H^+^-ATPase in stomatal guard cells. Plant Physiol. 178, 838–849. doi: 10.1104/pp.18.00544 30104254 PMC6181031

[B2] Ando E. Kollist H. Fukatsu K. Kinoshita T. Terashima I. (2022). Elevated CO_2_ induces rapid dephosphorylation of plasma membrane H^+^-ATPase in guard cells. New Phytol. 236, 2061–2074. doi: 10.1111/nph.v236.6 36089821 PMC9828774

[B3] Belin C. de Franco P. O. Bourbousse C. Chaignepain S. Schmitter J. M. Vavasseur A.. (2006). Identification of features regulating OST1 kinase activity and OST1 function in guard cells. Plant Physiol. 141, 1316–1327. doi: 10.1104/pp.106.079327 16766677 PMC1533939

[B4] Christie J. M. (2007). Phototropin blue-light receptors. Annu. Rev. Plant Biol. 58, 21–45. doi: 10.1146/annurev.arplant.58.032806.103951 17067285

[B5] Doi M. Kitagawa Y. Shimazaki K.-I. (2015). Stomatal blue light response is present in early vascular plants. Plant Physiol. 169, 1205–1213. doi: 10.1104/pp.15.00134 26307440 PMC4587438

[B6] Falhof J. Pedersen J. T. Fuglsang A. T. Palmgren M. (2016). Plasma membrane H^+^-ATPase regulation in the center of plant physiology. Mol. Plant 9, 323–337. doi: 10.1016/j.molp.2015.11.002 26584714

[B7] Gao C. (2021). Genome engineering for crop improvement and future agriculture. Cell 184, 1621–1635. doi: 10.1016/j.cell.2021.01.005 33581057

[B8] Harper J. F. Manney L. Sussman M. R. (1994). The plasma membrane H^+^-ATPase gene family in Arabidopsis: genomic sequence of AHA10 which is expressed primarily in developing seeds. Mol. Gen. Genet. 244, 572–587. doi: 10.1007/BF00282747 7969026

[B9] Haruta M. Tan L. X. Bushey D. B. Swanson S. J. Sussman M. R. (2018). Environmental and genetic factors regulating localization of the plant plasma membrane H^+^-ATPase. Plant Physiol. 176, 364–377. doi: 10.1104/pp.17.01126 29042459 PMC5761788

[B10] Hasan M. M. Corpas F. J. Fang X. W. (2022b). Light: a crucial factor for rhizobium-induced root nodulation. Trends Plant Sci. 27, 955–957. doi: 10.1016/j.tplants.2022.07.002 35840482

[B11] Hasan M. M. Gong L. Nie Z. F. Li F. P. Ahammed G. J. Fang X. W. (2021). ABA-induced stomatal movements in vascular plants during dehydration and rehydration. Environ. Exp. Bot. 186, 104436. doi: 10.1016/j.envexpbot.2021.104436

[B12] Hasan M. M. Liu X. D. Waseem M. Guang-Qian Y. Alabdallah N. M. Jahan M. S.. (2022a). ABA activated SnRK2 kinases: an emerging role in plant growth and physiology. Plant Signal. Behav. 17, 2071024. doi: 10.1080/15592324.2022.2071024 35506344 PMC9090293

[B13] Hasan M. M. Liu X. D. Yao G. Q. Liu J. Fang X. W. (2024). Ethylene-mediated stomatal responses to dehydration and rehydration in seed plants. J. Exp. Bot. 75, 6719–6732. doi: 10.1093/jxb/erae060 38367013

[B14] Hayashi Y. Fukatsu K. Takahashi K. Kinoshita S. N. Kato K. Sakakibara T.. (2024). Phosphorylation of plasma membrane H^+^-ATPase Thr881 participates in light-induced stomatal opening. Nat. Commun. 15 1194, 1–12. doi: 10.1038/s41467-024-45248-5 38378616 PMC10879185

[B15] Hayashi M. Inoue S. Takahashi K. Kinoshita T. (2011). Immunohistochemical detection of blue light-induced phosphorylation of the plasma membrane H^+^-ATPase in stomatal guard cells. Plant Cell Physiol. 52, 1238–1248. doi: 10.1093/pcp/pcr072 21666226

[B16] Hiyama A. Takemiya A. Munemasa S. (2017). Blue light and CO_2_ signals converge to regulate light-induced stomatal opening. Nat. Commun. 8 1284, 1–13. doi: 10.1038/s41467-017-01237-5 29101334 PMC5670223

[B17] Hõrak H. Sierla M. Tõldsepp K. Wang C. Wang Y. S. Nuhkat M.. (2016). A dominant mutation in the HT1 kinase uncovers roles of MAP kinases and GHR1 in CO_2_-induced stomatal closure. Plant Cell 28, 2493–2509. doi: 10.1105/tpc.16.00131 27694184 PMC5134974

[B18] Inoue S. Kinoshita T. (2017). Blue light regulation of stomatal opening and the plasma membrane H^+^-ATPase. Plant Physiol. 174, 531–538. doi: 10.1104/pp.17.00166 28465463 PMC5462062

[B19] Jakobson L. Vaahtera L. Tõldsepp K. Nuhkat M. Wang C. Wang Y. S.. (2016). Natural variation in Arabidopsis Cvi-0 accession reveals an important role of MPK12 in guard cell CO_2_ signaling. PloS Biol. 14, e2000322. doi: 10.1371/journal.pbio.2000322 27923039 PMC5147794

[B20] Kerbler S. M. Wigge P. A. (2023). Temperature sensing in plants. Annu. Rev. Plant Biol. 74, 341–366. doi: 10.1146/annurev-arplant-102820-102235 36854477

[B21] Kholodenko B. N. Okada M. (2021). Reengineering protein-phosphorylation switches. Science 373, 25–26. doi: 10.1126/science.abj5028 34210865 PMC8327301

[B22] Kinoshita T. Hayashi Y. (2011). New insights into the regulation of stomatal opening by blue light and plasma membrane H^+^-ATPase. Int. Rev. Cell Mol. Biol. 289, 89–115. doi: 10.1016/B978-0-12-386039-2.00003-1 21749899

[B23] Kinoshita T. Toh S. Torii K. U. (2021). Chemical control of stomatal function and development. Curr. Opin. Plant Biol. 60, 102010. doi: 10.1016/j.pbi.2021.102010 33667824

[B24] Kostaki K. I. Coupel-Ledru A. Bonnell V. C. Gustavsson M. Sun P. McLaughlin F. J.. (2020). Guard cells integrate light and temperature signals to control stomatal aperture. Plant Physiol. 182, 1404–1419. doi: 10.1104/pp.19.01528 31949030 PMC7054865

[B25] Li J. Guo Y. Yang Y. (2022). The molecular mechanism of plasma membrane H^+^-ATPases in plant responses to abiotic stress. J. Genet. Genomics 49, 715–725. doi: 10.1016/j.jgg.2022.05.007 35654346

[B26] Li S. Zhang J. Liu L. Wang Z. Li Y. Guo L.. (2020). SlTLFP8 reduces water loss to improve water-use efficiency by modulating cell size and stomatal density via endoreduplication. Plant Cell Environ. 43, 2666–2679. doi: 10.1111/pce.v43.11 32799324

[B27] Lu J. He J. Zhou X. Zhong J. Li J. Liang Y. K. (2019). Homologous genes of epidermal patterning factor regulate stomatal development in rice. J. Plant Physiol. 234, 18–27. doi: 10.1016/j.jplph.2019.01.010 30660943

[B28] Merlot S. Leonhardt N. Fenzi F. Valon C. Costa M. Piette L.. (2007). Constitutive activation of a plasma membrane H^+^-ATPase prevents abscisic acid-mediated stomatal closure. EMBO J. 26, 3216–3226. doi: 10.1038/sj.emboj.7601750 17557075 PMC1914098

[B29] Miao R. Russinova E. Rodriguez P. L. (2022). Tripartite hormonal regulation of plasma membrane H^+^-ATPase activity. Trends Plant Sci. 27, 588–600. doi: 10.1016/j.tplants.2021.12.011 35034860

[B30] Nishimura N. Sarkeshik A. Nito K. Park S. Y. Wang A. Carvalho P. C.. (2010). PYR/PYL/RACR family members are major *in vivo* ABI1 protein phosphatase 2C interacting proteins in Arabidopsis. Plant J. 61, 290–299. doi: 10.1111/j.1365-313X.2009.04054.x 19874541 PMC2807913

[B31] Palmgren M. G. (2001). Plant plasma membrane H^+^-ATPases: powerhouses for nutrient uptake. Annu. Rev. Plant Physiol. Plant Mol. Biol. 52, 817–845. doi: 10.1146/annurev.arplant.52.1.817 11337417

[B32] Praat M. De Smet I. van Zanten M. (2021). Protein kinase and phosphatase control of plant temperature responses. J. Exp. Bot. 72, 7459–7473. doi: 10.1093/jxb/erab345 34283227

[B33] Ren H. Park M. Y. Spartz A. K. Wong J. H. Gray W. M. (2018). A subset of plasma membrane-localized PP2C.D phosphatases negatively regulate SAUR-mediated cell expansion in Arabidopsis. PloS Genet. 14, e1007455. doi: 10.1371/journal.pgen.1007455 29897949 PMC6016943

[B34] Roelfsema M. R. Hedrich R. Geiger D. (2012). Anion channels: master switches of stress responses. Trends Plant Sci. 17, 221–229. doi: 10.1016/j.tplants.2012.01.009 22381565

[B35] Shahinnia F. Le Roy J. Laborde B. Sznajder B. Kalambettu P. Mahjourimajd S.. (2016). Genetic association of stomatal traits and yield in wheat grown in low rainfall environments. BMC Plant Biol. 16, 1–14. doi: 10.1186/s12870-016-0838-9 27378125 PMC4932692

[B36] Sinha R. Zandalinas S. I. Fichman Y. Sen S. Zeng S. Gómez-Cadenas A.. (2022). Differential regulation of flower transpiration during abiotic stress in annual plants. New Phytol. 235, 611–629. doi: 10.1111/nph.v235.2 35441705 PMC9323482

[B37] Takemiya A. Sugiyama N. Fujimoto H. Tsutsumi T. Yamauchi S. Hiyama A.. (2013). Phosphorylation of BLUS1 kinase by phototropins is a primary step in stomatal opening. Nat. Commun. 4 2094, 1–8. doi: 10.1038/ncomms3094 23811955

[B38] Toda Y. Wang Y. Takahashi A. Kawai Y. Tada Y. Yamaji N.. (2016). *Oryza sativa* H^+^-ATPase (OSA) is involved in the regulation of dumbbell-shaped guard cells of rice. Plant Cell Physiol. 57, 1220–1230. doi: 10.1093/pcp/pcw070 27048369 PMC4904443

[B39] Toh S. Inoue S. Toda Y. Yuki T. Suzuki K. Hamamoto S.. (2018). Identification and characterization of compounds that affect stomatal movements. Plant Cell Physiol. 59, 1568–1580. doi: 10.1093/pcp/pcy061 29635388

[B40] Toh S. Inoue S. Toda Y. Yuki T. Suzuki K. Hamamoto S.. (2021). Overexpression of plasma membrane H^+^-ATPase in guard cells enhances light-induced stomatal opening, photosynthesis, and plant growth in hybrid aspen. Front. Plant Sci. 12, 766037. doi: 10.3389/fpls.2021.766037 34899787 PMC8663642

[B41] Vahisalu T. Kollist H. Wang Y. F. Nishimura N. Chan W. Y. Valerio G.. (2008). SLAC1 is required for plant guard cell S-type anion channel function in stomatal signalling. Nature 452, 487–491. doi: 10.1038/nature06608 18305484 PMC2858982

[B42] Vu L. D. Xu X. Zhu T. Pan L. van Zanten M. de Jong D.. (2021). The membrane-localized protein kinase MAP4K4/TOT3 regulates thermomorphogenesis. Nat. Commun. 12 2842, 1–14. doi: 10.1038/s41467-021-23112-0 33990595 PMC8121802

[B43] Wang L. Chang C. (2024). Stomatal improvement for crop stress resistance. J. Exp. Bot. 75, 1823–1833. doi: 10.1093/jxb/erad477 38006251

[B44] Wang C. Hu H. Qin X. Zeise B. Xu D. Rappel W. J.. (2016). Reconstitution of CO_2_ regulation of SLAC1 anion channel and function of CO_2_-permeable PIP2;1 aquaporin as CARBONIC ANHYDRASE4 interactor. Plant Cell 28, 568–582. doi: 10.1105/tpc.15.00637 26764375 PMC4790870

[B45] Wang Y. Noguchi K. Ono N. Inoue S. I. Terashima I. Kinoshita T. (2014). Overexpression of plasma membrane H^+^-ATPase in guard cells promotes light-induced stomatal opening and enhances plant growth. Proc. Natl. Acad. Sci. U.S.A. 111, 533–538. doi: 10.1073/pnas.1305438111 24367097 PMC3890815

[B46] Wang T. Ye W. Wang Y. Zhang M. Aihara Y. Kinoshita T. (2021). Protease inhibitor-dependent inhibition of light-induced stomatal opening. Front. Plant Sci. 12, 735328. doi: 10.3389/fpls.2021.735328 34567048 PMC8462734

[B47] Wong J. H. Klejchová M. Snipes S. A. Nagpal P. Bak G. Wang B.. (2021). SAUR proteins and PP2C.D phosphatases regulate H^+^-ATPases and K^+^ channels to control stomatal movements. Plant Physiol. 185, 256–273. doi: 10.1093/plphys/kiaa023 33631805 PMC8133658

[B48] Xu X. Liu H. Praat M. Pizzio G. A. Jiang Z. Driever S. M.. (2024). Stomatal opening under high temperatures is controlled by the OST1-regulated TOT3–AHA1 module. Nat. Plants 11, 105–117. doi: 10.1038/s41477-024-01859-w 39613896

[B49] Yamauchi S. Takemiya A. Sakamoto T. Kurata T. Tsutsumi T. Kinoshita T.. (2016). The plasma membrane H^+^-ATPase AHA1 plays a major role in stomatal opening in response to blue light. Plant Physiol. 171, 2731–2743. doi: 10.1104/pp.16.01581 27261063 PMC4972258

[B50] Zhang M. Wang Y. Chen X. Xu F. Ding M. Ye W.. (2021). Plasma membrane H^+^-ATPase overexpression increases rice yield via simultaneous enhancement of nutrient uptake and photosynthesis. Nat. Commun. 12, 735. doi: 10.1038/s41467-021-20964-4 33531490 PMC7854686

[B51] Zhang X. Wang H. Takemiya A. Song C. P. Kinoshita T. Shimazaki K. (2004). Inhibition of blue light-dependent H^+^ pumping by abscisic acid through hydrogen peroxide-induced dephosphorylation of the plasma membrane H^+^-ATPase in guard cell protoplasts. Plant Physiol. 136, 4150–4158. doi: 10.1104/pp.104.046573 15563626 PMC535845

